# Initial immune response after exposure to *Mycobacterium tuberculosis* or to SARS-COV-2: similarities and differences

**DOI:** 10.3389/fimmu.2023.1244556

**Published:** 2023-08-17

**Authors:** Alessandra Aiello, Saeid Najafi-Fard, Delia Goletti

**Affiliations:** Translational Research Unit, National Institute for Infectious Diseases Lazzaro Spallanzani- Istituto di Ricovero e Cura a Carattere Scientifico (IRCCS), Rome, Italy

**Keywords:** SARS-CoV-2, *M. tuberculosis*, COVID-19, tuberculosis, innate response, T cell response, antibody response, co-infection

## Abstract

Tuberculosis (TB), caused by *Mycobacterium tuberculosis* (Mtb) and Coronavirus disease-2019 (COVID-19), whose etiologic agent is severe acute respiratory syndrome coronavirus-2 (SARS-CoV-2), are currently the two deadliest infectious diseases in humans, which together have caused about more than 11 million deaths worldwide in the past 3 years. TB and COVID-19 share several aspects including the droplet- and aerosol-borne transmissibility, the lungs as primary target, some symptoms, and diagnostic tools. However, these two infectious diseases differ in other aspects as their incubation period, immune cells involved, persistence and the immunopathological response. In this review, we highlight the similarities and differences between TB and COVID-19 focusing on the innate and adaptive immune response induced after the exposure to Mtb and SARS-CoV-2 and the pathological pathways linking the two infections. Moreover, we provide a brief overview of the immune response in case of TB-COVID-19 co-infection highlighting the similarities and differences of each individual infection. A comprehensive understanding of the immune response involved in TB and COVID-19 is of utmost importance for the design of effective therapeutic strategies and vaccines for both diseases.

## Introduction

Coronavirus disease-2019 (COVID-19), whose etiologic agent is severe acute respiratory syndrome coronavirus-2 (SARS-CoV-2) and tuberculosis (TB), that is caused by the bacterial pathogen *Mycobacterium tuberculosis* (Mtb), are the two-leading causes of death from a single infectious agent in humans. In the past 3 years, SARS-CoV-2 has been responsible for more than 7 million deaths, and Mtb for 4.5 million worldwide (1, 2).

SARS-CoV-2 is an enveloped RNA-based single-stranded virus recently emerged belonging to the Betacoronavirus genus. The first case of COVID-19 dates back to 2019 in Wuhan, China, and it is thought to be the result of a zoonotic spill-over event that likely occurred from bats and humans and finally caused the global pandemic (3). More than 700 million SARS-CoV-2 infections have been reported worldwide (to date, as of June 2023) (1). According to WHO, the largest number of confirmed cases are in Europe, Western Pacific and Americas (**Table 1**) (1). The spread of the virus was probably aided also by the onset of highly mutated forms of SARS-CoV-2, defined as “variants of concern” (VOCs), with enhanced transmission rate and with relatively lower morbidity and mortality compared to the ancestral strain (94, 95).

**Table 1:** Comparison of the features of SARS-CoV-2 and M. *tuberculosis* in terms of cell tropism, disease development and diagnosis.

Characteristics	COVID-19	Pulmonary TB disease
Etiologic agent	SARS-CoV-2	*Mycobacterium tuberculosis*
Epidemiology	Incidence rate in 2021: 206 million (Africa: 5 million; Americas: 66 million; Eastern Mediterranean: 12 million; Europe: 75.5 million; South-Est Asia: 32.7 million and Western Pacific: 10.7 million) Mortality in 2021: 3.5 million (1)	Incidence rate in 2021: 10.6 million (Africa: 2.46 million; Americas: 309.000; Eastern Mediterranean: 860.000; Europe: 230.000; South-Est Asia: 4.82 million and Western Pacific: 1.89 million) Mortality in 2021: 1.6 million (2)
Incubation period	2-14 days (average 5 days) (1)	From 8 weeks to a lifetime (2)
Time to develop a T cell specific response	From day 5 after infection (4, 5)	From 4-6 weeks on (6, 7)
Correlate of protective immune response	Neutralizing antibodies (8, 9)	Likely T cell-mediated response (10)
Route of transmission	Aerosols, droplets and contaminated surfaces (11–14)	Aerosols and droplets (15, 16)
Cell tropism	Primary targets: respiratory epithelial cells, such as ciliated cells, secretory goblet cells and alveolar epithelial type II cells within the nasal cavity and the upper and lower respiratory tract. Secondary targets: kidneys, small intestines, pancreas, blood vessels, testes and other tissues expressing ACE2 (3, 17, 18).	Primary target: alveolar macrophages, pneumocytes, epithelial cells (19–21) Secondary targets: lymph nodes, central nervous system, bones/joints, genitourinary tract, abdomen (intra-abdominal organs, peritoneum), and pericardium (22–25).
Entry mechanisms	Plasma membrane fusion, endocytic pathway, cell-to-cell transmission (26–28)	Phagocytosis (29, 30)
Main receptors	ACE2 as primary receptor and TMPRSS2 for the activation of the spike protein. Other receptors include integrins, neuropilin 1 (NRP1), phosphatidylserine receptors, the C-type lectins, asialoglycoprotein receptor 1 (ASGR1), Kringle Containing Transmembrane Protein 1 (KREMEN1), and CD147 (3, 26–28, 31).	Dectin-1, the complement receptor 3, TLRs, mannose receptor, the dendritic cell-specific intercellular adhesion molecule (ICAM)-3-grabbing nonintegrin (DC-SIGN), Fc receptors, scavenger receptors and CD14 (29, 30).
Innate immune response	Early production of type I IFN, IL-1β, IL-6, TNF-α and chemokines. Cytokine storm and late IFN-I production in severe COVID-19 patients (4, 5, 32–34). Neutrophilia, NET generation (35–38)	Early production of IL-1β, IL-1α, IL-6, TNF-α, IFN-γ and chemokines (21, 39). High monocyte/lymphocyte ratio (40)
Adaptive immune response	Lymphocytopenia, increased T cell activation, T cell dysfunctions, neutralizing antibodies (IgM, IgA and IgG) (41–51).	Lymphocytopenia, granuloma formation high T cell activation and finally exhaustion, antibody production (IgG) (52–58).
Detection tools for T cell response	IGRA, Flow cytometry Evaluated antigens: spike, N and M proteins/peptides (45, 59–63)	TST, IGRA, Flow cytometry Evaluated antigens: PPD, ESAT-6, CFP-10, Ag85 B, HBHA, Rv2628, MTB300 proteins/peptides (2, 6, 56, 64–68).
Main evasion mechanisms	Autoantibodies against IFN-I, mutations in spike protein (32, 69–75).. The envelope (E) protein down-regulates the CD1d, an antigen-presenting molecule of invariant NKT (iNKT) cells, and suppresses these cells (76).	Inhibition of phagosome maturation, induction of TLR2 antagonist glycolipids, NET formation for Mtb replication, and suppression of the production of pro-inflammatory cytokines or release of anti-inflammatory cytokines (77–84).
Clinical manifestation	Cough, fatigue, fever, sneezing, runny nose, sore throat, and anosmia in the first few days followed by shortness of breath, diarrhea, vomiting etc. (1, 85)	Cough, fatigue, fever, weight loss, night sweats, chest pain and hemoptysis (2, 86).
Comorbidities may influence clinical outcome	Old age, hypertension, diabetes, biological therapy based on CD20 inhibitors (1, 85, 87–89).	HIV, diabetes, malnutrition, biological therapy based on TNF-α inhibitors, extreme age (children below 5 age or elderly) (2, 86, 87, 90).
Diagnostics	RT-PCR or rapid antigenic tests (1, 91).	Microscopy, culture, molecular tests such as Gene-Xpert, and chest X-ray (2, 92, 93).
Samples	Naso- and -oropharyngeal swabs and saliva (1, 91)	Sputum or bronchoalveolar lavage (2, 92, 93)

SARS-CoV-2, severe acute respiratory syndrome coronavirus 2; COVID-19, coronavirus disease-19; Mtb; Mycobacterium tuberculosis; ACE2, angiotensin-converting enzyme 2; TMPRSS2, type 2 transmembrane serine protease; TLR, toll-like receptor; N, nucleocapsid; M, membrane; IFNs, interferons; IL, interleukin; TNF, tumor necrosis factor; NET, Neutrophil extracellular traps; Ig, immunoglobulin; IGRA, IFN-γ release assay; TST, tuberculin skin test; PPD, purified protein derivative; ESAT-6, early secretory antigenic target; CFP-10, 10-kDa culture filtrate protein; HBHA, heparin-binding hemagglutinin antigen.

On the contrary, Mtb is an ancient slow growing bacterium that has plagued the human population for thousand years. To date, it is estimated that one third of the world population is infected with Mtb (2), and about 5-10% of the Mtb-exposed and -infected individuals will progress to TB disease. In most of them bacilli are detectable in the sputum (15). According to WHO, the largest number of confirmed cases are in Africa and South-Est Asia (**Table 1**) (2).

Although Mtb and SARS-CoV-2 are distinct pathogens, they share several features summarized in **Table 1**. The main transmission route for both pathogens is *via* droplets (> 100 µm particles) or aerosols (< 100 µm particles) that are expelled by an ill individual by coughing, sneezing, talking, and breathing (11, 16). These particles can travel short distances in the air before being inhaled (12). However, for SARS-CoV-2, the infection can also occur as a result of contact with contaminated surfaces or objects on which virions can persist even for 72 hours (13, 14). Regarding Mtb, infection can also occur during autopsies (96) or during the spill of caseus material, i.e. from a scrofula when the cervical tuberculous lymphadenitis drains the material outside (97–99).

**Figure 1:**
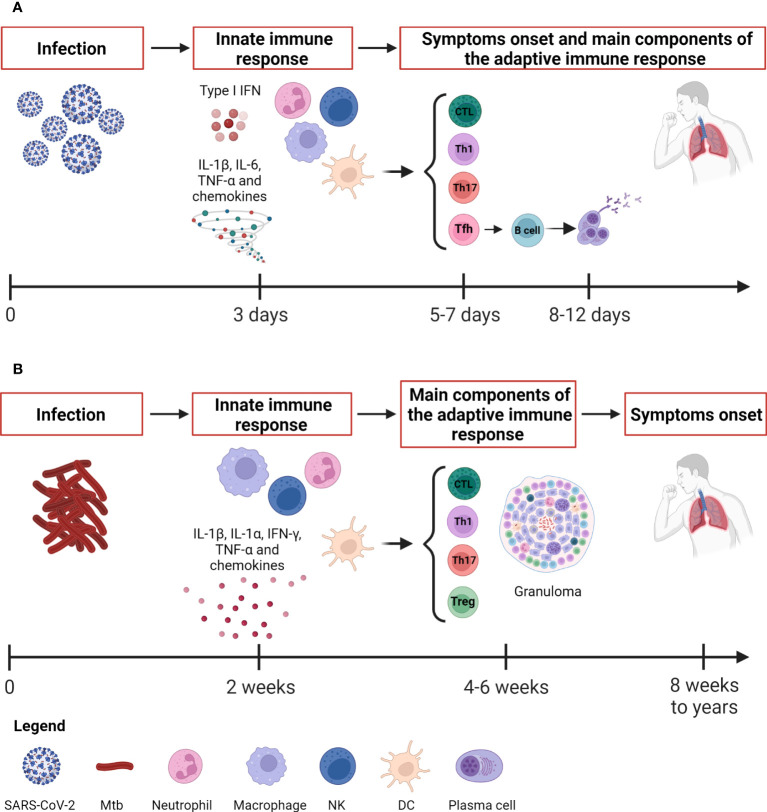
Kinetic of the immune response to SARS-CoV-2 and Mtb. **(A)** SARS-CoV-2 infection evolves rapidly. The innate immune response occurs after about 3 days and is detectable through immunoenzymatic assays and flow cytometry. The antigen-specific T cell response appears around 5-7 days concurrently also with the onset of symptoms, whereas the antibody response appears later around 8-12 days. The adaptive immune response is detectable by immunoenzymatic assays, flow cytometry and IGRA. **(B)** Mtb causes a slow-progressing infection that might result in the development of TB disease even after many years. The innate immune response occurs after about 2 weeks and is detectable through immunoenzymatic assays and flow cytometry as for SARS-CoV-2. The antigen-specific T cell response is detectable around 4-6 weeks by means IGRA, TST, immunoenzymatic assays and flow cytometry. SARS-CoV-2, severe acute respiratory syndrome coronavirus 2; Mtb, *Mycobacterium tuberculosis*; IFNs, interferons; DCs, dendritic cells; NK, natural killer; Tfh, T follicular helper lymphocytes; Th, T helper; IGRA, IFN-γ release assay; TST, tuberculin skin test. Created with BioRender.com.

While SARS-CoV-2 shows a short incubation period (2-14 days) before symptoms onset, in Mtb infection it can range from eight weeks to a lifetime (1, 2) (**Table 1** and **Figure 1**).

Considering the route of transmission, it is not surprising that both SARS-CoV-2 and Mtb firstly infect the respiratory system causing symptoms such as cough, fatigue and fever. In addition, SARS-CoV-2-infected subjects also experience sneezing, runny nose, sore throat, and anosmia in the first few days followed by shortness of breath, diarrhea, vomiting, etc. (85), whereas in TB patients weight loss, night sweats, chest pain and coughing up of blood were reported (86). This similarity in symptoms might make the diagnosis difficult; however, in most cases the COVID-19 symptoms are short-lived compared to those of TB, which has a long incubation with long-lasting symptoms duration.

Both agents can be detected in respiratory samples such as nasopharyngeal swab or saliva for SARS-CoV-2, and sputum or bronchoalveolar lavage (BAL) for Mtb.

The diagnosis can require different tools. For SARS-CoV-2 infection, molecular swab is the first choice in case of suspected symptomatic individuals, contacts of confirmed cases with symptoms and for the screening of health workers. In other contexts, it is recommended to use rapid antigenic tests that are less labor-intensive and costly and can provide results in less than half an hour (91) (**Table 1**).

Regarding Mtb, two main types of tests are used to determine the traditionally called latent infection, now defined “tuberculosis infection” (2): the tuberculin skin test (TST) and interferon (IFN)-γ release assays (IGRA). For patients with suspected pulmonary TB, the Center for Disease Control (CDC) recommends performing an acid-fast-bacilli smear on three different sputum specimens (92). Moreover, Gene-Xpert (Cepheid, Sunnyvale, CA, USA) is a widely accepted diagnostic test for TB detection in direct smear negative cases (93).

Notably, SARS-CoV-2 and Mtb-infected individuals show a diverse spectrum of clinical manifestations. Patients infected with SARS-CoV-2 can experience a clinical outcome ranging from asymptomatic to mild/moderate infection up to severe disease (particularly with Wuhan strain and in those not vaccinated), which can also progress to acute respiratory distress syndrome (ARDS) (1). Indeed, SARS-CoV-2 can interfere with the host immune system leading to hyperinflammatory state, immune dysregulation, and extensive lung damage (100, 101).

Differently, Mtb-exposed individuals remain clinically asymptomatic due to the development of an immune response that controls Mtb replication (102, 103). It has been shown that some individuals heavily exposed to Mtb can clear the infection early before the emergence of the adaptive immune response, can keep a negative score to the TST and IGRA, and therefore do not show any evidence of infection (104). The lack of a detectable adaptive immune response in these resistant individuals suggests the key role mediated by the local innate immunity. The difficulty of treating and eradicating Mtb is related to the ability of the mycobacteria to survive and replicate within human cells.

In both infections, the clinical manifestations may be more severe in presence of comorbidities. In this regard, they share similar risk factors in terms of comorbidities as advanced age (87), and diabetes (90), although they have specific peculiarities as hypertension and biological therapy with CD20 inhibitors for COVID-19 and HIV infection, malnourishment and biological therapy based on TNF-α inhibitors for TB (90) (**Table 1**).

An effective and timely immune response plays a pivotal role in affecting the clinical course of both COVID-19 and TB. This review aims to provide an overview of innate and adaptive immune responses induced after the exposure to Mtb and SARS-CoV-2 highlighting the similarities and differences of each individual infection and their crosstalk in TB-COVID-19 co-infection.

## Cell tropism and entry mechanisms

Viral entry is the first and pivotal step for the viral life cycle. Not surprisingly, blocking virus entry is a primary target of several therapeutic strategies to prevent the subsequent steps and inhibit viral replication and host cell pathology (3). Although both SARS-CoV-2 and Mtb are airborne pathogen entering *via* droplets, and primarily infect the human respiratory system, they differ by cellular tropism and entry mechanisms.

### SARS-CoV-2 and cell tropism and entry mechanisms

SARS-CoV-2 has a broad spectrum of tropism. The human angiotensin-converting enzyme 2 (ACE2) represents the major cellular entry point for the virus, thus the expression of ACE2 defines which tissues can be potentially infected by SARS-CoV-2 (3, 26). The epithelial cells such as subset of ciliated cells, secretory goblet cells and alveolar epithelial type II cells within the nasal cavity and the upper and lower respiratory tract, represent the primary targets for the initial infection and spread of SARS-CoV-2. In this regard, the higher amount of viral RNA was found in ciliated and epithelial progenitors (105). Interestingly, although the human respiratory tract is the main target for the virus due to its airborne transmissibility, ACE2 expression in kidneys and gastrointestinal tract is even higher than the lungs (17). Notably, extrapulmonary organs such as the kidneys, small intestines, pancreas, blood vessels, testes and other tissues can be additional targets for SARS-CoV-2, thus explaining the variety of symptoms associated to the infection (17, 18).

**Figure 2:**
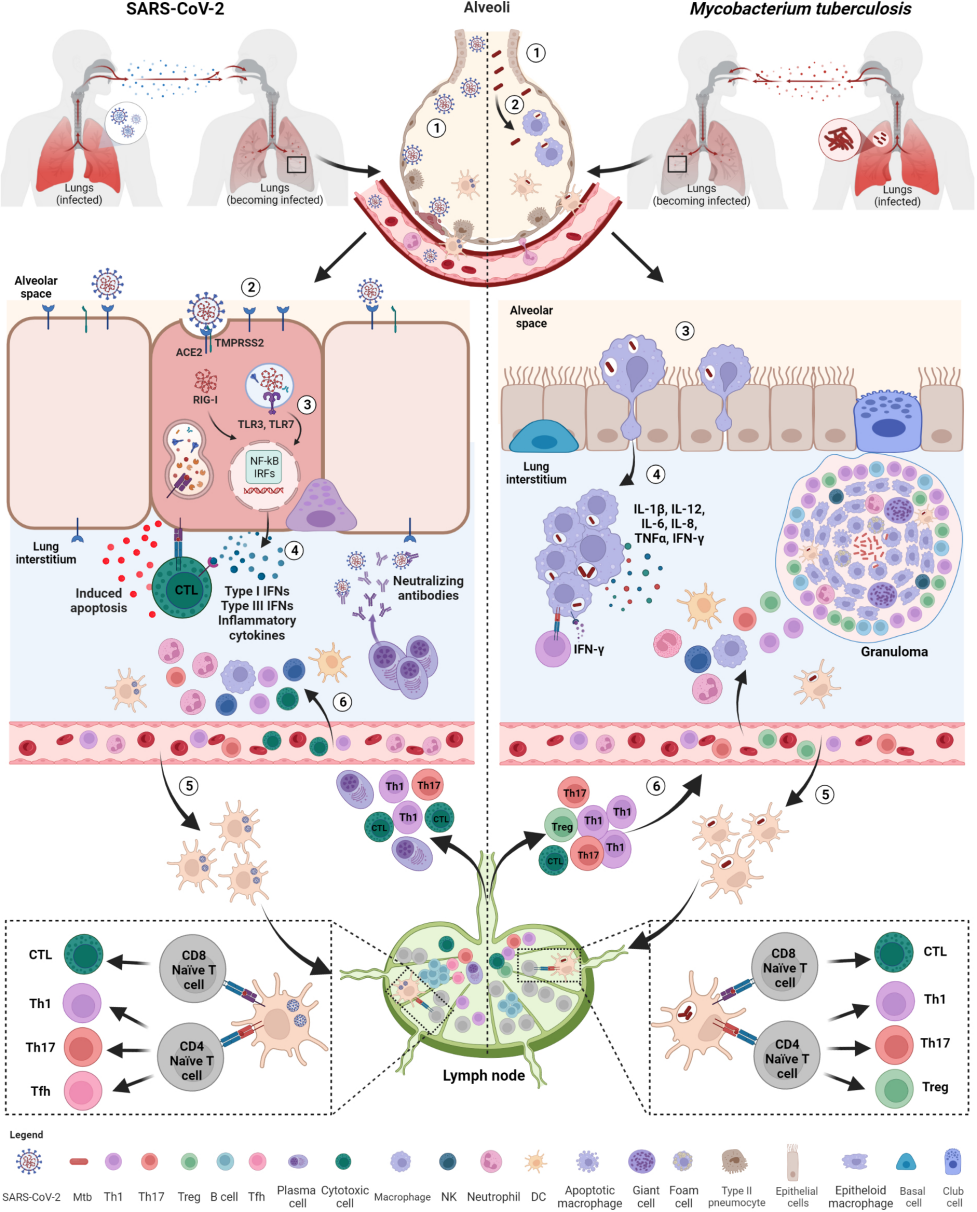
Initial immune response after exposure to SARS-CoV-2 and Mtb. Both SARS-CoV-2 and *M. tuberculosis* (Mtb) are transmitted by aerosols or droplets. SARS-CoV-2 infection (1): virions enter into the airways and (2), once arrived in the lung, infect epithelial lung cells *via* recognition and binding of the spike protein to the ACE2 cell receptor. (3) Viral RNA, once released inside the cells, is recognized by endosomal (TLR3, TLR7) or cytosolic (RIG-I) receptors and activate downstream signaling pathways (NF-kB and IRFs) (4) leading to the release of IFNs, pro-inflammatory cytokines and chemokines favoring immune cell recruitment, including neutrophils and DCs. (5) Infected DCs migrate to the lymph nodes for T and B cell priming. (6) Primed T cells and plasma cells go back to the infection site *via* blood where they exert their functions, including apoptosis induced by cytotoxic T cells and viral neutralization. Mtb infection: (1) Mtb bacilli enter into the airways and (2) are phagocytosed by alveolar macrophages. (3) Alveolar macrophages migrate to lung interstitium, where they form aggregates and (4) release cytokines promoting the recruitment of immune cells, such neutrophils, macrophages and DCs. (5) Infected DCs migrate to lymph nodes to prime T cells that are recruited at the infection sites where they contribute to the formation of the organized granuloma. SARS-CoV-2, severe acute respiratory syndrome coronavirus 2; Mtb, *Mycobacterium tuberculosis*; ACE2, angiotensin-converting enzyme 2; TMPRSS2, type 2 transmembrane serine protease; TLR, toll-like receptor; IFNs, interferons; RIG, retinoic acid-inducible gene-I; NF-kB, nuclear factor kappa-light-chain-enhancer of activated B cells; IRFs, interferon regulatory factors; DCs, dendritic cells; NK, natural killer; Tfh, T follicular helper lymphocytes; Th, T helper. Created with BioRender.com.

SARS-CoV-2 gains access to cells mainly through two possible routes, the plasma membrane fusion and the endocytic pathway. The entry route used by the virus is dependent on the expression of cell surface proteases, which are needed for the activation of the viral protein (27, 28), and it is primarily mediated by the structural protein spike, a trimeric glycoprotein that binds to the ACE2 (26). After binding, spike undergoes a conformational change that allows the proteolytic cleavage before membrane fusion (**Figure 2**).

Spike activation can occur either at the cell surface or in endosomes and consists of two different proteolytic events. The first proteolytic event occurs during spike biosynthesis and it is mediated by the host pro-protein convertase furin that cleaves the polybasic S1/S2 junction (106) generating the two subunits S1 and S2 non-covalently linked and with different roles in the viral entry (107). The amino-terminal S1 subunit includes a receptor-binding domain (RBD) that is involved in the initial recognition of ACE2 receptor (108), whereas the carboxy-terminal S2 presents highly conserved regions that catalyse the fusion between viral and host cell membranes, crucial to release the viral RNA genome and start the replication in the target cell. A further cleavage at the S2’ site is needed to expose the S2’ fragment, a highly hydrophobic fusion peptide that starts the fusion of membranes (109, 110).

Interestingly, TMPRSS2, which is a type 2 transmembrane serine protease (TTSPs) expressed in the human upper and lower respiratory tract, heart, prostate and gastrointestinal tracts (111–113), has been shown to prime spikes on cell surface thus allowing the entry *via* membrane fusion (26). In the absence or insufficient availability of cell surface proteases, in particular TMPRSS2, SARS-CoV-2 prefers to enter *via* clathrin-mediated endocytosis (114). In this case, the conformational modifications of the spike occur in the acidic environment of endosomes and its cleavage is mediated by the members of the cathepsin family (e.g. B and L). While the virus takes 10 minutes to enter the cells *via* cell surface membrane fusion, the pH-dependent endocytosis process needs about 40–60 minutes after infection (28).

The cleavage of S1/S2 can have an impact on viral fitness and transmission, thus affecting viral infectivity (115). Notably, during the COVID-19 pandemic, several mutations have accumulated in S1 and S2 subunits of the spike causing the emergence of several SARS-CoV-2 VOCs capable of escaping the immune system, while preserving the steps of activation of the spike protein. The different infectivity rate in the epithelial cells of the nose, bronchi, and lung by SARS-CoV-2 VOC is correlated with the different protease expression, subsequent transmissibility, and severity of disease (18, 87, 116–118).

Emerged Omicron subvariants are less dependent on TMPRSS2-mediated spike activation at the plasma membrane, showing a reduced replication of the virus in the lung and intestinal cultures, while a similar replication rate was observed in the nasal epithelia compared to the Delta variant (117, 119, 120). Likely, this modified tropism allowed a major air transmission of the virus, in accordance with the highest rate of spread observed in the latest variants compared with the ancestral one (69). Moreover, the different spike protease tropism resulted in the diminished pathogenesis in the lung.

Besides TMPRSS2, other TTSPs or metalloproteases can mediate SARS-CoV-2 entry. For instance, TMPRSS2 and TMPRSS4 promote viral entry into human enterocytes of the proximal digestive tract (121), and matrix metalloproteases (MMPs), such as ADAM10 and ADAM17, seem to be involved in the cleavage at the S2 site in cells lacking TMPRSS2 (122–124). Moreover, coagulation factors, such as factor Xa and thrombin, can directly cleave spike protein at both cleavage sites and thus further contributing to infection at the stage of viral entry (125, 126).

Furthermore, other molecules have been suggested as alternative receptors for the SARS-CoV-2 entry process including integrins, neuropilin 1 (NRP1), phosphatidylserine receptors, the C-type lectins, asialoglycoprotein receptor 1 (ASGR1), Kringle Containing Transmembrane Protein 1 (KREMEN1), and CD147, as reviewed by Jackson and colleagues (27, 31).

Notably, SARS-CoV-2 could also infect cells through other mechanisms that allow the virus to escape the immune recognition favoring its spread in the host. In this regard, SARS-CoV-2-infected cells can directly fuse with adjacent cells expressing ACE2 through S1/S2 cleaved SARS-CoV-2 spikes resulting in the formation of multinucleated cells or syncytia (127, 128). The syncytia formation favors a cell-to-cell transmission of the virus without even the need to assemble viral particles or to release the virus in the extracellular environment (129). SARS-CoV-2-induced multinucleated pneumocytes and syncytia formation is a feature of severe COVID-19 patients, suggesting their involvement in the COVID-19 pathogenesis (130–132). Moreover, these structures might cause direct cytopathic effects to lymphocytes. In this regard, Zhang and colleagues reported that lymphocytes could be internalized by syncytia by forming cell-in-cell structures and leading to cell death (133).

Another possible mechanism for viral entry is mediated by extracellular vesicles (EVs) containing particles or viral components well documented in SARS-CoV-2-infected cells (134).

Regardless of the mechanism and molecules involved in SARS-CoV-2 entry, the virus replicates triggering the host immune response.

### M. tuberculosis and cell tropism and entry mechanisms

As for SARS-CoV-2, the first interactions between bacteria and host occur in the lungs after the inhalation of the aerosolized Mtb. The size of Mtb droplets (2–5 µm particles) is important to ensure the passage through the upper respiratory tract into the alveolar space, where bacilli primarily encounter pneumocytes, epithelial cells (AEC), and alveolar macrophages (AMs) with anti-bacterial capacities (19–21). On the other hand, larger droplets can be stuck in the upper airways or oropharynx probably explaining the onset of the extrapulmonary forms of TB localized in the oropharynx but lacking evidence of concurrent pulmonary disease (135).

Once entered into the airways, Mtb is phagocytosed by AMs, which are permissive for infection establishment. In the upper airway, Mtb invades the specialized epithelial cells called microfold cell (M cell) through the binding to the scavenger receptor B1 in both mouse and human tissue (136, 137). Similar to SARS-CoV-2, Mtb can disseminate to other organs including the lymphatics and lymph nodes that are the main sites of extrapulmonary TB (22). Lymphatic endothelial cells, the adipose tissue and the bone marrow have been identified as extrapulmonary niches where Mtb may persist for long time (23–25).

The receptors involved in the Mtb entry into cells have not been fully demonstrated. Phagocytosis of Mtb by macrophages seems not occur *via* a single receptor-mediated pathway, but rather it seems to be mediated by multiple receptors including dectin-1, the complement receptor 3, mannose receptor, the dendritic cell-specific intercellular adhesion molecule (ICAM)-3-grabbing nonintegrin (DC-SIGN), Fc receptors, scavenger receptors and CD14 (29). Other receptors, such as Toll-like receptors (TLRs) are involved in the recognition of mycobacteria. To enable their entrance into AMs, mycobacteria exploit a group of pathogen-associated molecular patterns (PAMPs) expressed on its surface, including mycobacterial lipoproteins such as the 19 kDa surface antigen LpqH, which acts as an adhesin playing a crucial role in both host-pathogen interactions and pleiotropic immune regulation through the engagement of the TLR1/TLR2 (30). The downstream signaling and the phagosomal fate depend on the type of receptor engaged during the phagocytosis.

Macrophages containing Mtb then migrate from the air space to the lung interstitium in an IL1-R signaling- and ESX-1 secretion system-dependent manner (138, 139). This is the first step preceding the formation of the granuloma, the pathologic hallmark of TB (**Figure 2**).

## Innate immune response

Whereas Mtb causes a slow-progressing infection that might result in the development of TB disease even after many years, the SARS-CoV-2 infection evolves rapidly causing COVID-19 (**Figure 1**). Within the immunological response to Mtb and SARS-CoV-2, both the innate and adaptive responses play an important role. The innate immune response is a nonspecific response that serves as initial defense against pathogens. It consists of humoral components (cytokines, chemokines, interferons, complement and coagulation-fibrinolysis systems, and naturally occurring antibodies) and cellular components (natural killer cells, macrophages, dendritic cells and other innate lymphocytes). Innate immunity aids in controlling the infection, in the identification and eradication of infected cells as well as in the development of the adaptive immunity (59, 140).

### Innate immune response to SARS-CoV-2

The heterogeneous course of SARS-CoV-2 infection depends on the immune response at the early stages of infection (141). Considering the rapid course of COVID-19, the capability of patients with asymptomatic or mild disease to control the infection is likely due to the innate immune response since the adaptive response occurs days later, with the T cell immunity preceding the B cell response occurring after 2 weeks (**Figure 1**).

Early on, an effective control of SARS-CoV-2 spread depends on the induction of a robust antiviral response and on the ability of alveolar macrophages to eliminate the virus and the infected cells through phagocytosis.

Immune cells resident within the lung recognize SARS-CoV-2 through several pathogen-recognition receptors (PRRs), such as TLRs (TL3 and TLR7), retinoic acid-inducible gene I (RIG-I)-like receptors (RLRs), nucleotide-binding oligomerization domain (NOD)-like receptors (NLRs) and inflammasomes. As a result, downstream signaling pathways involving nuclear factor kappa-light-chain-enhancer of activated B cells (NF-kB) and interferon regulatory factors (IRFs) are activated inducing the production of multiple pro-inflammatory cytokines such as IL-1β, IL-6, and TNF-α, several chemokines (CCL20, CXCL1, CXCL2, CXCL3, CXCL5, CXCL6, CXCL8 and CXCL16) (32, 33) and antiviral IFNs resulting in the initial inflammation. The local innate immune response attracts and activates into the site of infection further innate immune cells such as neutrophils, monocytes, dendritic cells (DCs), natural killer (NK), and innate lymphoid cells aimed to promote viral clearance (142) (**Figure 2**). Consequently, the combined action of innate immune cells, cytokines, and chemokines may have an impact on the outcome of SARS-CoV-2 infection (143).

Although SARS-CoV-2 induces a pro-inflammatory state, there are reports of reduced IFN release (70, 144); in fact, SARS-CoV-2 is more effective at suppressing IFN responses compared to other respiratory viruses (71). Type I IFN, which includes IFN-α and IFN-β, represents the primary defensive response against viral infections by the induction of antiviral effector molecules encoded by IFN-stimulated genes (ISGs) and immunomodulatory responses (145). In SARS-CoV-2-infected individuals, the presence of a quick type I IFN production soon after infection contributes to protection against critical illness as observed in studies conducted in individuals exposed to COVID-19 cases (4, 5, 34).

On the contrary, if a strong and rapid antiviral response is lacking, the ongoing infection can lead to an exuberant release of cytokines and chemokines that is amplified by the further infiltration of circulating immune cells, finally provoking the so-called “cytokine storm”, which can be caused by infectious and non-infectious agents, and which in COVID-19 is responsible for the immunopathology associated with its severe presentation (141). Based on the evidence, individuals with highly compromised IFN-I response, which means no IFN-β and low IFN-α production and activity due to neutralizing auto-antibodies or inherited errors of type I IFN immunity, do not control the primary SARS-CoV-2 infection and they are more at risk of fatal COVID-19 (70, 146–148). Moreover, a low number and an impaired functionality of plasmacytoid dendritic cells (pDCs), which are the main IFN producers, have been found in bronchoalveolar lavage fluid (BALF) from severe or critical patients compared to the moderate ones (149). Also, a lower frequency of circulating pDCs was found in samples from SARS-CoV-2-infected individuals than in controls (150).

*In vitro* studies have shown the presence of a huge amount of NF-kB-dependent proinflammatory mediators in BALF (CCL2, CCL3, CCL4, and CXCL10) (151) and in circulation (IP-10, IL-6, and IL-8, IL-1, IFN-γ, IL-17, TNF-α, MCP-1, G-CSF, GM-CSF, IL-1RA, CCL2, CCL3, CCL5, CCL8, CXCL2, CXCL8, CXCL9, and CXCL16) (32, 60, 70, 152–155).

Patients with COVID-19 generally show migration of neutrophils and monocytes into the nasopharyngeal mucosa in response to chemokines released by infected epithelial cells (e.g. CXCL1, CXCL3, CXCL6, CXCL15, CXCL16, and CXCL17) (156).

Once reached the lung, neutrophils as phagocytes may exert a protective role in the clearance of the infection by secreting leukotrienes, reactive oxygen species (ROS), and forming neutrophil extracellular traps (NET), which are aggregates of extracellular DNA, histones, microbicidal proteins and proteases aimed to entrap and kill pathogens. However, neutrophils are known to be implicated in COVID-19 pathology as hyperinflammation drivers through increased cytokine production and cell degranulation (35). Indeed, their extensive and prolonged activation causes an hyperinflammatory environment and cellular infiltrations that may result in the tissue damage observed in the ARDS and increased mortality (36, 37). Indeed, a high neutrophil-to-lymphocyte ratio (NLR), that is a marker of inflammation and infection, and NET DNA complexes have been found in severe COVID-19 compared with mild/moderate cases or healthy controls (38).

In addition to NET generation, another source of hyperinflammation associated with COVID-19 is the activation of the NLRP3-inflammasome due to the interaction of the nucleoprotein (N) with NLRP3 (157). In this regard, a study conducted in an ACE2 humanized mouse model of COVID-19 showed that, in response to infection, macrophages activate inflammasomes causing the release of IL-1β and IL-18 and undergo pyroptosis, thus favoring the pathogenesis of acute lung injury (158).

During SARS-CoV-2 infection, monocytes/macrophages are involved either as virus target or as producer of inflammatory cytokines and undergo phenotypical changes (159). Alterations in the phenotype of monocytes consisting of reduced antigenic presentation and dysregulated immune response have been observed (35). In the peripheral blood of COVID-19 patients there are cell subsets of mixed M1/M2 macrophages secreting IL-6, TNF-α and IL-10 and characterized by higher expression of CD80 and CD86 (35, 160–162).

NK cells are innate lymphocytes that are recruited along with macrophages and neutrophils in the lungs as confirmed by the analysis on BALF samples of COVID-19 patients (163). NK cells usually exert an antiviral activity through the production of the effector cytokines IFN-γ and TNF-α and limit tissue fibrosis (164). Regarding the protective role of NK cells against infection, Witkowski and colleagues reported that SARS-CoV-2-infected individuals with a higher NK cell number at hospitalization showed a more rapid clearance of viral load (165). Although during early stages of infection NK cells may contribute to control viral replication and dissemination, their migration in affected tissue may favor the enhancement of inflammation. In this context, a reduced peripheral cell count and functional impairment of NK cells with an enhanced expression of the cytolytic proteins perforin and granzyme B have been found in patients with severe COVID-19 (166–168).

CD1d-restricted NKT cells are other types of innate lymphocytes that are involved in antiviral immunity (169). To counteract their function, the envelope (E) protein of SARS-CoV-2 reduces the expression of the antigen-presenting molecule CD1d thus inhibiting the activation of innate NKT cells and enhancing SARS-CoV-2 virulence (76).

The activation of the innate immune system is essential to mount an effective adaptive immune response. In this regard, DCs, as professional antigen presenting cells (APCs), represent a point of junction between innate and adaptive immune response as they migrate to lymph nodes to activate naïve T lymphocytes (170).

### Innate immune response to M. tuberculosis

The innate immune response to Mtb infection is multifaceted with several different cell types and functions involved. Upon pattern recognition, a variety of cellular functions, including phagocytosis, autophagy, and apoptosis will be launched by the host to clear or control Mtb (171–173). In particular, macrophages with antimicrobial mechanisms such as nitric oxide synthesis and antimicrobial peptides such as cathelicidin represent the first defense line against Mtb infection (174).

The investigation of the early events and host responses against Mtb in humans is very challenging and difficult as the progression of infection is generally slow and individuals often do not know the exact time of exposure or infection (175). Therefore, a validated model that recapitulates TB in human lungs is critical to support TB research. In this regard, a number of *in vitro* systems (176), spheroids (177), human airway organoids (178), and experimental animal models of TB such as zebrafish (179), mouse (180), guinea pig (181), rabbit (182) and rat (183) have provided new insights into the local events that occur during few days and weeks post Mtb infection. In particular, Mtb infection in nonhuman primates closely recapitulates human TB and these models can be used to study the full spectrum of infection outcome and pathology of TB (184).

Early in infection, the infected cells are activated and start to release some early mediators of inflammation such as TNF-α, IL-1α, IL-1β, IFN-γ and chemo-attractant molecules (e.g. CXCL5, CXCL8), some of which also characterize the early stages of SARS-CoV-2 infection (**Figure 2**). These soluble factors mediate the recruitment to the site of infection of different blood cell types including neutrophils, monocytes, macrophages and DCs (21, 39), which are necessary for starting early granuloma formation (139). These innate granulomas include cells that are not yet fully activated, thus favoring the dissemination of mycobacteria from infected macrophages to uninfected cells.

Notably, the EVs released from infected cells containing mycobacterial components, including lipoarabinomannan, the Ag85 complex and lipoproteins, have been shown to contribute to the migration of immune cells to the lungs (185). Moreover, EVs can modulate immune response by promoting the release of proinflammatory cytokines and by increasing autophagy and superoxide production (185, 186).

During the first 10 days post-infection, Mtb almost exclusively resides and replicates inside AMs, suggesting that these cells provide an early niche for Mtb growth (139, 187, 188). In a murine model of TB, Mtb was reported to be equally distributed between AMs, DCs and neutrophils 14 days post-aerosol challenge (189).

As already mentioned for SARS-CoV-2 and also known for other infections including Mtb, DCs play a crucial role by transporting bacteria from the site of infection to the draining lymph nodes (64) in order to prime naïve T cells and start an adaptive immune response (190, 191). An involvement of CCR2^+^ inflammatory monocytes in the Mtb delivery to pulmonary lymph nodes has also been reported (192). Notably, a higher monocyte/lymphocyte ratio is observed in Mtb-infected patients (40).

Neutrophils are other professional phagocytes that have been shown to be involved in the early innate immune response against Mtb through a direct antimicrobial activity and chemokines/cytokines production (193). They readily phagocytose Mtb and can destroy it *via* ROS, proteases and antimicrobial peptides (AMPs). They can also undergo apoptosis and microbe-containing apoptotic neutrophils can be phagocytosed by macrophages and DCs and then transported to the lymph nodes (194, 195).

In addition, mucosal-associated invariant T cells (MAITs) are a group of T cells restricted to a nonclassical molecule MR-1 and not to the classical major histocompatibility complex (MHC) molecules. MAITs are also involved in the early responses to Mtb by producing IFN-γ and TNF-α, and showing cytotoxic activity upon recognition of microbe-derived riboflavin metabolites (196).

Moreover, there is evidence for a role of NK cells in controlling Mtb infection, by killing the pathogen through antibody-dependent cellular cytotoxicity, directly targeting the Mtb by binding to cell wall components such as mycolic acid, arabinogalactan, peptidoglycan through receptors including TLR-2, NKp44, NKp46, and NK group 2D (NKG2D), promoting the maturation of phagolysosome and phagocytosis by producing cytokines such as IFN-γ and TNF-α and by killing Mtb-infected macrophages through the release of granules (perforin, granulysin, and granzyme) (175, 197–199). However, it is not well known whether the role of NK cells is as important as that of macrophages or cytokines such as IFN-γ or TNF-α.

Nonetheless, Mtb has evolved several strategies to evade the host’s immune system through its unique cell wall structure, intracellular survival, dormancy and the ability to modulate immune response. Mtb has adapted to survive and replicate in macrophages by inhibiting phagosome maturation (77–80) and promoting necrosis over apoptosis (200). Several types of programmed necrosis in response to Mtb infection, such as inflammasome-mediated pyroptosis and NET-associated NETosis have been identified (201–203). However, NETosis may facilitate the interactions between neutrophils and other immune cells rather than killing Mtb directly (81). Moreover, the formation of NETs can be induced *via* type I IFN signaling to favor MTB replication (82). Mtb inhibits also innate immune response by induction of TLR2 antagonist glycolipids (83). It also modulates the immune response through the release of molecules that suppress the production of proinflammatory cytokines or even by inducing the production of anti-inflammatory cytokines (84).

As for SARS-CoV-2, the control of Mtb infection requires a timely innate response as well as an effective adaptive response.

## Adaptive immune response

The adaptive immune response comprises antibody and cell-mediated responses and takes approximately 2 to 3 weeks before we can measure it (59). It is involved in the specific recognition of pathogens and in the establishment of the immunological memory. Notwithstanding the importance of innate responses, a coordinated cellular immunity is crucial for disease control in both SARS-CoV-2 and Mtb infection.

### T cell response to SARS-CoV-2

In the majority of cases, SARS-CoV-2 infection induces adaptive antigen-specific responses, viral clearance and immunological memory finally resulting in an asymptomatic or mild disease. However, a failure of the first line defense mechanisms, particularly of innate IFN, may act as triggering factor for viral proliferation and immune dysregulation. Indeed, the delayed/ineffective adaptive responses and exaggerated inflammatory response can promote immunopathogenesis of COVID-19, particularly ARDS (204–207).

Several lines of evidence from both human studies and animal model systems have shown that an effective T cell response is required to control and eradicate SARS-CoV-2 infection by releasing cytokines and other anti-inflammatory factors (208).

During the infection, subepithelial DCs present SARS-CoV-2-specific peptides through MHC class I and II molecules on the cell surface, thus promoting the activation of CD8^+^ and CD4^+^ T cells, respectively, which migrate to the lung after antigen exposure. Indeed, the lung is characterized by the presence of tissue-resident T cells with a memory phenotype (CD69^+^, CD103^+/-^, CD45RA,CCR7^-^) originated from the priming of naïve T cells (209). Interestingly, an involvement of EVs in the regulation of antigen presentation and T cell activation has also been reported (210).

While CD8^+^ T cells recognize and kill the infected cells, CD4^+^ T cells contribute to activate B cells for antibody secretion and CD8^+^ T cells to exert the cytotoxic activity, and to produce cytokines that favor immune cell migration at the site of infection (143).

Initial studies conducted by Grifoni and colleagues, and subsequently confirmed by others showed CD4^+^ and CD8^+^ viral specific T cell responses in most infected individuals mainly against spike antigen, although present also against other structural (nucleocapsid and membrane proteins) and non-structural SARS-CoV-2 antigens (61–63). Since spike protein has been identified as the most immunogenic antigen, it has been employed for many of the currently used SARS-CoV-2 vaccines (61, 62).

Unlike Mtb infection, the early development of antigen-specific T cell responses is generally observed within 7 days after the onset of COVID-19 symptoms, peaks at 14 days and may be detectable even if SARS-CoV-2 specific antibodies are lacking (5) (**Figure 1**). Several studies have shown that asymptomatic or pauci-symptomatic individuals are characterized by a strong SARS-CoV-2-specific CD4^+^ T cell response (41, 211–213). Surprisingly, CD4^+^ T cell responses were also observed in 40% to 60% of unexposed individuals likely because of the cross-recognition between SARS-CoV-2 and other “common cold” coronaviruses (63).

T cell activity has been associated with a less disease severity (8, 59). The critical role played by T cells in the protection against the severe disease has been highlighted also with the occurrence of different VOCs with an increased ability to escape neutralizing antibodies (214–216). Indeed, the spike-specific T cell response induced by both vaccination and natural infection seems to be not affected by the amino acid mutations that characterize the VOCs, including Omicron, in healthy subjects and in the vulnerable populations (217–221). Indeed, the availability of thousands of SARS-CoV-2 epitopes that may be recognized by T cells makes unlikely that the virus may successfully escape the T cell response by mutating the epitopes.

SARS-CoV-2 infection mainly support the differentiation of CD4^+^ T lymphocytes toward T helper 1 (Th1), T helper 17 (Th17) and T follicular helper (Tfh) cells (**Figure 2**).

An appropriate Th1 immune response is necessary for protection against COVID-19, as an early and rapid expansion of IFN-γ-secreting SARS-CoV-2-specific T cells was detected over the course of acute infection and was associated with viral clearance (42, 222) and mild disease (43, 223, 224).

Chauss and colleagues showed that asymptomatic SARS-CoV-2-infected individuals present in the BALF CD4^+^ T cells switched from a predominantly pro-inflammatory Th1 phenotype toward an IFN-γ and IL-10-producing phenotype that enable them the viral control without causing pathology (225). The mechanism behind the switching phenotype is triggered by cell-intrinsic complement that orchestrates an autocrine/paracrine autoregulatory vitamin D (VitD) loop to initiate Th1 shutdown. During this process, Vitamin D induces epigenetic changes in the CD4^+^ T cells and recruits transcriptional factors, including c-JUN, STAT3 and BACH2 finally resulting in the switch off of Th1 programs and in the IL-10 induction (225). In patients with severe COVID-19 these regulatory processes are lacking and thus exacerbated Th1 cytokine profiles are prevail (226).

The lack of a fine-tuned Th1 immune response can cause an exacerbated reaction that precedes cytokine storm promoting the differentiation of Th2 cells that are related to a poor prognosis (227). In this regard, Gil-Etayo and colleagues observed in COVID-19 patients a significant reduction in the percentage of Th1 and Th17 cells whereas a higher frequency of activated Th2 cells. Moreover, a higher number of senescent Th2 cells together with higher levels of IL-15 were observed in patients with a fatal outcome (227).

In addition, Th17 cells are strongly activated in severe COVID-19, thus favoring cell-mediated immunopathology through the production of IL-17 and GM-CSF (44). IL-17 released by Th17 cells induces the activation of monocytes/macrophages, DCs, and neutrophils which, in turn, increases the release of cytokines (IL-1, IL-6, IL-8, IL-21, TNF-α, and MCP-1), thus promoting the cytokine storm (44).

It has been reported that the polarization of CD4^+^ T cells toward Th17 instead of Th1 can be promoted by neutrophils as well as by the up-regulation of pro-inflammatory cytokines IL-1β, IL-6 and IL-23 (228).

Tfh cells are localized within the germinal centers of the secondary lymphoid organs and they are primarily involved in the activation and proliferation of B cells, and the production of high affinity antibodies (59) as well as in the assistance of CD8^+^ T cell functions (229).

In rhesus macaques CD8^+^ T cells are crucial for viral clearance especially when a reduced humoral response is present (230). In this regard, a weak CD8^+^ T cell response has been associated with a poor prognosis (45, 231). Indeed, a delayed or lacking CD8^+^ T cell response was found in patients with severe or fatal outcomes probably due to the inability of T cells to rapidly limit viral replication (59).

Besides CD4^+^ and CD8^+^ T cells, regulatory T cells (Treg) have been shown to play a critical role in SARS-CoV-2 infection, particularly as regulators of the inflammatory response. Perturbations in Treg phenotype, such as the reduced expression of Foxp3 and cytokines including IL-10 and TGF-β, have been associated with disease severity (232).

Quantitative and/or functional deficiency of T cells is associated with pathological processes responsible for tissue damage. Indeed, a characteristic hallmark of severe COVID-19 is the peripheral lymphopenia accompanied by a reduced count of monocytes, eosinophils, basophils, but not neutrophils (46). Possible explanations for T cell depletion is the SARS-CoV-2 infection of T cells through the binding of the spike protein to the CD147 or CD26 expressed on cell surface (233), their recruitment to infected site, or their apoptosis *via* Fas/Fas ligand or TNF (234–237). Furthermore, increased levels of IL-6, IL-10 and TNF-α may contribute to lymphopenia (47, 238). The prolonged peripheral lymphocytopenia increases the risk of secondary bacterial infections (88). Also, an immunosuppression following hyperinflammation in COVID-19 disease has been described, in particular NLRs and TLRs were shown to be associated to immunosuppression (239).

A reduced number of peripheral Treg cells has also been observed in severe cases of COVID-19, likely leading to the development of lung pathology (232).

As COVID-19 progresses, a different T cell functionality has also been observed. Early during the acute phase of SARS-CoV-2 infection, T lymphocytes are characterized by a highly activated cytotoxic phenotype, whereas in convalescent individuals they show a polyfunctional and memory phenotype (41, 44, 47, 240). CD8^+^ T cells expressing markers of exhaustion such as PD-1^+^ TIM3^+^ increase over the infection and this scenario seems to be related to IL-10 blood levels. The hyper-activation of T cells along with the dysfunctionality of DCs and Tregs may increase the overwhelming alveoli inflammation and cytokine storm in COVID-19 (241).

In light of what is reported in literature, an efficient T cell response is fundamental for viral clearance.

### Antibody response to SARS-CoV-2

The antibody response usually appears by 1-2 weeks later than SARS-CoV-2 specific T cell response that is detectable 5-6 days post-infection (4, 5) (**Figure 1**). Within few days post-infection, B cells are rapidly activated in extrafollicular foci to differentiate in short-lived plasma cells that predominantly produce IgM antibodies but also IgG or IgA-switched to initially stem viral infection, while waiting for the production of antibodies with higher affinity. The first IgM, IgA and IgG are measurable in the sera between 8 and 12 days after symptom onset (48). Subsequently, within the germinal centers in the secondary lymphoid organs, antigen-specific B cells undergo somatic hypermutation and isotype-switching resulting in the production of high-affinity IgG antibodies that mainly recognize nucleocapsid and spike proteins (242, 243). Cross-sectional and longitudinal studies showed that Enzyme-linked immunosorbent assay (ELISA) titers and neutralizing antibodies are detectable around 14 days after symptom onset, peak in 3 to 4 weeks, and decline subsequently causing a reduction of protection and increasing the risk of SARS-CoV-2 re-infection (9, 49, 50, 244).

However, it has been observed that anti-RBD antibodies, neutralizing activity and RBD-specific memory B cells are mostly stable between 6 and 12 months after infection (245, 246), likely owing to the presence of a long-lived plasma cell compartment located in the bone marrow (247–249).

The protective role of the antibodies is limited to those specific for the viral spike protein because they neutralize the virus by hindering the binding between spike and ACE2 receptor and thus blocking its entry, and by promoting effector functions *via* the binding to the complement and Fc receptors (250).

In the case of neutralizing antibodies, the engagement of Fc receptors can potentiate neutralization (251, 252). Non-neutralizing antibodies may promote antibody-dependent cellular cytotoxicity (ADCC) and antibody-dependent cellular phagocytosis (ADCP). In this regard, high ADCC activities are detected mainly in hospitalized patients and showed a kinetic similar to antibody titers with a peak at 2-4 weeks post-infection followed by a gradual decline (253–255).

Most of the antibodies are directed against epitopes localized in the receptor-binding motif (RBM) within the RBD of spike, whereas a minority is directed against the N-terminal domain (NTD) (256–258). Anti-NTD antibodies have less neutralizing activity than anti-RBD antibodies and they may act by interfering with the conformational changes necessary for fusion or binding to receptors such as transmembrane lectins DC-SIGN, L-SIGN and SIGLEC1 (259, 260).

The antibody response, either qualitative and quantitative, is dependent on the amount of the antigen and on the activity of the germinal centers. In this regard, patients with severe COVID-19 show higher titers of total and neutralizing antibodies than mild or asymptomatic patients, likely due to the stronger antigen response (261, 262). On the other hand, individuals undergoing B-cell depleting therapies, such as anti-CD20, show an impaired antibody response that is associated with a more severe course of COVID-19 (263).

While circulating antibodies may help to control viral dissemination within the host, mucosal antibodies such as the dimeric form of IgA that is secreted in the upper respiratory tract, play an important role in preventing the transmission of SARS-CoV-2, present a stronger neutralizing activity than circulating antibodies, and contribute to protection against re-infection (51, 264). Indeed, SARS-CoV-2 specific IgA have been found in saliva samples collected from infected individuals (249).

During SARS-CoV-2 infection, also autoantibodies targeting self-antigens, including type I IFN, were identified in some COVID-19 patients, particularly in those with a severe disease that are characterized by a reduced IFN production, as mentioned above (32, 70, 71). COVID-19 patients are also characterized by changes in B-cell subpopulations. In particular, increased number of proliferating, metabolically hyperactive plasma blasts and reduction of memory B cells have been found in patients with severe disease, whereas they disappeared with convalescence (261, 265, 266).

Nonetheless, SARS-CoV-2 has evolved different strategies to escape the immune response. Unlike bacteria such as Mtb, RNA viruses are usually characterized by high mutation rates. SARS-CoV-2 exploits this ability to accumulate mutations in the spike protein in order to avoid the immune recognition by neutralizing antibodies and to increase its transmissibility (69). In particular, the emerging VOCs has accumulated mutations mainly located in the RBM, in part due to the pressure exerted by the host immune system. It has been proposed that the concurrent onset of multiple mutations in the spike protein might occur during the prolonged infection in immunocompromised patients resulting in the emergence of variant strains (72, 73). These mutations increased affinity of the virus for the ACE2 receptor and improved its ability to evade the neutralizing antibody response induced by natural infection or following vaccination with the spike protein derived from the ancestral strain (74, 75).

Altogether, the humoral response has been shown to play a crucial role in the host immune protection against SARS-CoV-2 together with the T cell response.

### T cell response to M. tuberculosis

The infected monocytes, macrophages and DCs are thought to be key elements leading to Mtb dissemination and granuloma formation (39, 267). The infected professional antigen-presenting DCs travel to the lung draining lymph nodes where priming of naïve CD4^+^ and CD8^+^ T cells is initiated (52, 64, 190, 191, 268). Priming is a critical step for the initiation of the adaptive immunity that is crucial to hinder bacilli dissemination and control the infection. However, the adaptive (T cell) response takes longer to appear in infected hosts because Mtb or its antigens are transported late into the lymph nodes for T cell priming (269). In mice this occurs within 2-3 weeks post-infection (64, 65, 270), but in humans and non-human primates Mtb-specific T cell response in the periphery, measured as a response to TST, or IGRA, is usually not detectable until 4–6 weeks post-infection (6, 7).

It was found that Mtb-infected DCs in the lymph node are capable to release soluble and intact Mtb antigens that can be caught by uninfected DCs and efficiently presented to naïve CD4^+^ T cells to optimize CD4^+^ T cell priming and to initiate the adaptive immune response (271). Surprisingly, the capacity of Mtb-infected DCs in activation and proliferation of naïve Mtb-specific CD4^+^ T cells in the murine lymph node was found to be impaired likely due to lower MHC class II-peptide presentation by these infected APCs (189).

The primed T (and likely B) lymphocytes can then move to the site of infection and contribute to the formation of the organized granuloma that consists of modified macrophages as epithelioid cells and multinucleated giant cells accompanied by neutrophils and DCs in the center, infiltrated immune cells including granulocytes, antigen-specific T cells and few B cells in the periphery, with variable degrees of fibrosis or central caseous necrosis (**Figure 2**) (272, 273). Although the mechanisms driving protection and pathology within the granuloma microenvironments are still poorly understood, such mechanisms can be very important for the prognosis, and outcome of the disease (52).

Notably, granuloma structure and function protect the host from the dissemination of the infection, but it is also a way to facilitate the persistency of the infection (274). In fact, sterilizing immunity following Mtb infection is rare and even in the presence of a robust adaptive immune response to Mtb, the nature of the granulomas as well as the immune escape mechanisms of Mtb can restrict the host immune response to reliably eliminate the infection. This leads to develop a controlled infection, traditionally called latent infection, in most infected individuals. Mtb can survive in a dormant (non-replicating) state favored by hypoxic conditions inside solid granulomas that makes it difficult to be detected by the immune system (53, 275).

Within the granuloma, Mtb antigens persistently stimulate immune cells leading to immune activation, chronic inflammation, and finally cell exhaustion (54). Different T cell types and functions can exert a beneficial or even detrimental role. The peripheral localization of T cells restricts their access to the central core of the granuloma, where Mtb-infected macrophages reside, and this can limit the interactions between macrophages and lymphocytes. Moreover, a Mtb-induced immunosuppressive environment has been indicated in the granuloma in which IL-10 impairs Th1 activity and lysis of infected macrophages (276).

The important role for T-cell immunity and particularly IFN-γ-producing Th1 in controlling Mtb infection has been demonstrated in humans (10) and animal models (277, 278). IFN-γ is a key factor involved in CD4^+^ T cell-mediated protection by increasing autophagy and promoting phagosome maturation in macrophages (79) inducing the production of antimicrobial peptides (279), and limits the accumulation of non-protective CD4^+^ T cells in the lung vasculature (280).

In humans, HIV infection appears to be an important risk factor for TB disease progression likely due to CD4^+^ T cell depletion (10, 90). Also, depletion of CD4^+^ T cells in cynomolgus macaques with acute Mtb infection leads to exacerbated disease in most animals (278). Moreover, TB disease increases HIV replication, *in vivo* and *in vitro* through a mechanism of immune activation (281, 282).

The activation and proliferation of antigen-specific naïve CD4^+^ T cell subsets strongly depends on the cytokine milieu released by APCs. Particularly, macrophages are the main source of IL-1β, IL-6, IL-18, TNF-α, IL-10, and TGF-β, while DCs are the main producers of IL-12, IL-23, IL-27 and IFN-β (39). For instance, IL-12 produced by DCs differentiates naïve CD4^+^ T cells to Th1 which promote activation of the cell-mediated immunity needed to counteract intracellular pathogens (55). These cells secrete pro-inflammatory cytokines such as IL-2, IFN-γ and TNF-α to activate macrophages and cytotoxic CD8^+^ T cells (283). TNF-α is known to be necessary for the formation of a well-organized granuloma and host protection, as confirmed by the higher risk of developing TB disease and disseminated infection in subjects who underwent anti-TNF-α treatment (284, 285).

Activated cytotoxic CD8^+^ T cells and macrophages kill and eliminate pathogens and infected host cells by cytotoxic effector molecules such as perforin, granzymes and granulysin and by death receptor/ligand ligation (286).

Furthermore, IL-23 produced by DCs drives differentiation and functionality of Th17 cells that produce IL-17 which is a cytokine involved in neutrophil recruitment (287). IL-17 signaling appears to be essential for recruiting neutrophils to the site of infection early after Mtb infection in murine models (288), but a dysregulated production of this cytokine was also found to be associated with immunopathology driven by excess neutrophil recruitment and inflammation (289, 290).

Although inflammation is required for an effective immune response against harmful pathogens, the balance between pro- and anti-inflammatory cytokines is critical to control the disease and lung damage during Mtb infection (56, 291). Anti-inflammatory cytokines such as IL-4, IL-5, IL-13 released by Th2 cells and IL-10 and TGF-β by regulatory T cells are needed to suppress inflammation during immune response. However, these cells may promote long-term persistence of Mtb by favoring active immunosuppression rather than the expected tissue repair response (292).

In patients with TB disease, TST positive, *in vitro* PPD stimulation induced the production of IL-10, IFN-γ, and cell proliferation, whereas in those TST-negative PPD induced IL-10 but not IFN-γ release, without cell proliferation (293).

Altogether, a better understanding of the dynamically balanced immune response is fundamental for therapeutic strategies and subsequently for vaccine development.

### The role of B cells and antibodies in TB

Although Mtb infection induces strong antibody responses, the role of antibodies and B cells in TB has not been fully elucidated. Previous studies on B cell depletion have failed to definitively establish a role for these cells or antibodies in Mtb infection and control, although recent studies have demonstrated potentially protective roles of antibodies in humans and non-human primates (NHPs) after intravenous bacille Calmette-Guérin (BCG) vaccination (294, 295).

It has been shown that TB disease is associated with decreased B cell count and function compared with individuals who are infected with Mtb but without any clinical symptoms, suggesting that TB patients may be less able to develop successful antibody responses against Mtb (296–298).

Moreover, distinct glycosylation patterns on the Fc part of the antibodies (296), and isotype skewing to less potently immune-activating variants like IgG4 have been considered for this altered functional response (298, 299).

Surprisingly, heavily Mtb-exposed individuals who “resisted” to infection showed higher antibody functionality compared to those with TB infection, indicating an important role of antibodies in early protective immunity (300, 301).

Studies have shown that the interaction of Mtb with macrophages can be affected by antibodies in a variety of ways (57, 58). For instance, bacterial opsonization may alter vesicular trafficking and macrophage signaling. Moreover, the binding of antibodies to Fc receptors (activator or inhibitory) on macrophages can modulate their function (58).

Together, data suggest that B cells and antibodies may play an important role in protective immunity against mycobacterial infections; however, the diversity of antibody functions, the heterogeneity of the humoral immune response to Mtb, as well as the complexity of the interactions between B cells and other immune cells have been indicated as the major challenges to understand the impact of the humoral immune system in the immune protection at each stage of Mtb infection (58).

## M. tuberculosis and SARS-CoV-2 co-infection

Information on TB-COVID-19 co-infection in humans is still limited. Co-infection was reported around 1% in the Philippines (302), 5% in South Africa (303), and between 0.37% and 4.47% in China (304). Recent works suggest that TB-COVID-19 co-infection is associated with elevated risk of unfavorable clinical outcome, with a longer time to recovery, treatment failure, loss to follow-up rates, and higher rates of mortality compared to patients with COVID-19 alone (89, 305–308).

However, mechanistic studies are needed to understand the interactions during Mtb and SARS-CoV-2 dual infections, their effect on the host immune response and clinical outcomes. Understanding the early events and pathophysiology of TB-COVID-19 co-infection is warranted to find better ways to manage such cases, particularly in the high TB endemic areas. The dysregulated immune response induced by each pathogen can lead to an unbalanced inflammatory response, which can promote the progression and worsening of both diseases.

To date, the immune response for each pathogen has been well studied, whereas the impact of Mtb and SARS-CoV-2 co-infection on the innate and adaptive immune response, their crosstalk and cumulative impact on disease outcome in humans still need to be delineated (309–313).

In fact, the studies available have mostly focused on the clinical features of co-infected patients, characterizing a marked lymphopenia and increased levels of some markers of inflammation, such as C-reactive protein (CRP), D-dimer, ferritin, and describing the lung tissue damages (308, 312, 314, 315).

There are few published studies either *in vitro*, ex-vivo using human samples from co-infected individuals or animal models evaluating the immune response and immunopathology in the context of co-infection (**Table 2**).

**Table 2:** Studies that evaluated the immunopathology and the immune response in the context of M. tuberculosis and SARS-CoV-2 co-infection.

Study (ref)	Model	Immunological findings
Sheerin et al., 2023 (316)	*In vitro* model of infection with Mtb and SARS-COV-2 using human cells from HC	Characterizing distinct and overlapping immunological responses generated by SARS-CoV-2, Mtb, or during co-infection.
Hildebrand et al., 2022 (317)	*In vivo* animal model (mice) infected with Mtb and/or SARS-CoV-2; Uninfected controls	In lungs and spleen of co-infected mice: **↓** type 1 (IFN-γ, TNF-α), **↑** type 2 (IL-4 and IL-13) transcripts
Rosas Mejia et al., 2022 (318)	*In vivo* animal model (mice) infected with Mtb and/or SARS-CoV-2; Uninfected controls	In lungs of co-infected mice: **↓** IFN-γ, IL-6, IL-1β, and transcripts of IFN-γ, TNF-α, **↑** IL-10.
Rajamanickama et al., 2021 (319)	*In vitro* model using human cells from asymptomatic COVID-19 and TBI- asymptomatic COVID-19	In TBI+/SARS-CoV-2 IgG+: **↑** IgM, IgG, IgA, neutralizing antibodies against SARS-CoV-2 compared to TBI-/IgG+. **↑** proinflammatory cytokine/chemokines (IFN-γ, IL-2, TNF-α, IL-1α,IL-1β, IFN-α, IFN-β, IL-6, IL-12, IL-17, GM-CSF, CCL3, CXCL10) and anti-inflammatory cytokines (IL-4, IL-10, IL-25, and IL-33) compared to TBI-/IgG+.
Rajamanickam et al., 2022 (320)	*In vitro* model using human cells from asymptomatic COVID-19 with or without TBI	In TBI+/SARS-CoV-2 IgG+: **↑** baseline and Mtb-induced (but not mitogen) levels of IFN-γ, IL-2, TNF-α, IL-17A, IL-1β, IL-6, IL-12, CCL1, CXCL1, CXCL9, CXCL10, IL-4, IL-13. **↓** levels of IL-5 and IL-10 compared to TBI-/IgG+.
Musso et al., 2021 (315)	*In vitro* model using human cells from TB-COVID-19	Cell anergy in response to Mtb antigens and mitogen stimulation.
Petrone et al., 2021 (310)	*In vitro* model using human cells from COVID-19; TB-COVID-19; TBI-COVID-19; NO COVID-19	In TB-COVID-19 co-infected patients: **↓** specific IFN-γ response to SARS-CoV-2 compared to TBI-COVID-19 and COVID-19-only.
Najafi-Fard et al., 2023 (313)	*In vitro* model using human cells from TB-COVID-19; COVID-19; TB; HC	In co-infected patients: **↑** TNF-α, MIP-1β, and IL-9 compared with COVID-19-only. **↑** TNF-α, IL-1β, IL-17A, IL-5, FGF-basic, and GM-CSF compared with TB-only. **↓** specific response to SARS-CoV-2 and Mtb.
Riou et al., 2021 (311)	*In vitro* model using human cells from patients with or without COVID-19 co-infected or not with TB	In co-infected patients: **↓** SARS-CoV-2-specific and Mtb-specific CD4+ T cell responses with poor polyfunctional cell potentials.
du Bruyn et al., 2023 (314)	*In vitro* model using human cells from patients with or without COVID-19 co-infected or not with TB and/or HIV-1; HC	Comparable frequency of SARS-CoV-2-specific CD8+ T cell response between TB-COVID-19 co-infected and COVID-19-only patients.

COVID-19, CoronaVirus Disease 19; TB, tuberculosis; HC, healthy control; SARS-CoV-2, Severe acute respiratory syndrome coronavirus 2; Mtb, Mycobacterium tuberculosis; IFN, interferon, TNF, tumor necrosis factor; IL, interleukin; MIP, macrophage inflammatory protein; FGF, fibroblast growth factor; GM-CSF, granulocyte-macrophage-colony-stimulating factor; TBI, tuberculosis infection; Ig, immunoglobulin; CCL, Chemokine (C-C motif) ligand; Chemokine (C-X-C motif) ligand.

*In vitro* studies were recently performed by Sheerin and colleagues using a single-cell RNA-seq (scRNA-seq) approach to analyze the results from a co-infection performed using a whole blood platform (24 or 96 hours) from healthy adults. The authors characterized different and overlapping immunological responses generated by SARS-CoV-2 (ancestral strain) and Mtb (lineage 4 laboratory strain H37Rv) when a single infection or co-infection occurs. Based on marker gene expression, they identified 13 distinct clusters of cells showing diverse proportions of monocytes, T cells and neutrophils between different conditions and timepoints. The co-infected condition showed the major immune activation effect early (24h) post-infection with 238 immunological pathways uniquely enriched, including IFN-γ and TNF production, while 182 shared pathways were overlapping at 96h post-infection among different conditions. In contrast to SARS-CoV-2-only infection that caused extensive cell death by 96h post-infection, Mtb-only and co-infected conditions maintained monocyte, T cell and NK cell signatures, and negative regulation of the signaling of extrinsic apoptosis (316).

Interesting animal studies evaluating the impact of aerosol Mtb and SARS-CoV-2 co-infection in transgenic (K18-hACE2) C57BL/6 mice showed that pre-infection with Mtb resulted in lower SARS-CoV-2 viral loads at the lung tissue level, likely mediated by the heightened immune microenvironment of the lungs. In addition, after SARS-CoV-2 superinfection, increased bacterial loads in Mtb-infected tissues and decreased histiocytic inflammation were found. Moreover, SARS-CoV-2 caused a decreasing trend in type 1 (IFN-γ and TNF-α) and an increasing trend in type 2 (IL-4 and IL-13) cytokine transcript levels in Mtb-infected mice. These findings, which are usually associated with disseminated Mtb infection, suggest that SARS-CoV-2 may have a deleterious effect on TB outcome (317) through the immune dysregulation, potentially resulting in granuloma collapse and the subsequent Mtb dissemination (311).

Using two concomitant murine models of COVID-19 (SARS-CoV-2 infection of K18-hACE2 mice and mouse-adapted SARS-CoV-2 [MACoV2] infection of C57BL/6 mice) it was shown that chronically Mtb H37Rv-infected mice were resistant to the pathological consequences of secondary SARS-CoV-2 infection, and SARS-CoV-2 infection did not affect Mtb burdens. Single-cell RNA sequencing of the lungs of the co-infected animals showed that resistance could be due to T and B cells expansion upon viral challenge. Interestingly, lower lung protein levels of IFN-γ, IL-6 and IL-1β as well as mRNA levels of IFN-γ and TNF-α and higher levels of IL-10 were found in co-infection than in Mtb-monoinfection at the 30 days post-infection (318), similar to Hildebrand and colleagues (317)

Regarding the evaluation of the immune responses in co-infected humans, two studies have demonstrated that Mtb infection can modulate humoral (antibody) and cytokine responses to SARS-CoV-2 infection (319) and *vice versa* (320) in investigations conducted in TB endemic countries. Rajamanickam and colleagues demonstrated that individuals seropositive (IgG^+^) for SARS-CoV-2 infection and with TB infection (TBI^+^/SARS-CoV-2 IgG^+^) were characterized by higher levels of specific antibodies (IgM, IgG and IgA) and neutralizing antibodies against SARS-CoV-2 compared to individuals with only SARS-CoV-2 infection. Moreover, elevated plasma levels of proinflammatory cytokine/chemokine responses including IFN-γ, IL-2, TNF-α, IL-1α, IL-1β, IFN-α, IFN-β, IL-6, IL-12, IL-17, GM-CSF, CCL3, CXCL10 and anti-inflammatory cytokines such as IL-4, IL-10, IL-25 and IL-33 were found in TBI^+^/SARS-CoV-2 IgG^+^ subjects. These results show that Mtb infection can modulate the immune responses in asymptomatic SARS-CoV-2-infected individuals (319). In an additional study, it was shown that TBI^+^/SARS-CoV-2 IgG^+^ individuals have higher baseline and Mtb-induced (but not mitogen) levels of several pro- and anti-inflammatory cytokines/chemokines including IFN-γ, IL-2, TNF-α, IL-17A, IL-1β, IL-6, IL-12, CCL1, CXCL1, CXCL9, CXCL10, IL-4, IL-13 and reduced levels of IL-5 and IL-10 compared to TBI^-^/SARS-CoV-2 IgG^+^ individuals. These findings suggest modulating effects of SARS-CoV-2 infection on the immune responses of individuals with Mtb infection (320). However, these results were obtained in TB-infected individuals with only asymptomatic SARS-CoV-2 infection and the influence of each pathogen on the disease severity and the outcome of each infection were not evaluated.

Differently, clinical outcome was assessed in a case of multidrug-resistant (MDR)/TB-COVID-19 co-infected patient affected by bilateral cavitary pulmonary TB, that subsequently developed COVID-19-associated pneumonia which led to a fatal outcome. Death was probably due to the immuno-suppressed state of the patient, as shown by the low lymphocyte count and by the lack of response to Mtb antigens and mitogen (315).

In addition, a cohort of TB-COVID-19 co-infected patients with different severity of COVID-19 showed a reduced ability to mount a specific immune response to SARS-CoV-2 stimulation compared to patients with TBI and COVID-19 (TBI-COVID-19) or with COVID-19 only (310). In particular, in TB-COVID-19 co-infected patients TNF-α, MIP-1β, and IL-9 showed significant elevated levels compared to COVID-19 only, and TNF-α had the highest discriminant power. Moreover, TNF-α, IL-1β, IL-17A, IL-5, FGF-basic, and GM-CSF were increased in co-infected compared to patients with TB-only. Importantly, co-infection was associated with an impairment of SARS-CoV-2-specific and a reduced Mtb-specific immune response (313).

In agreement with these results, Riou and colleagues demonstrated in TB-COVID-19 co-infection impaired SARS-CoV-2-specific and Mtb-specific CD4^+^ T cells with reduced polyfunctional cell potentials, proliferation cell capacity, and augmented cell activation markers (311). However, the frequency of SARS-CoV-2 specific CD8^+^ T cell response to peptides spanning the M, N and S sequences in TB-COVID-19 co-infected patients was found to be comparable with patients with COVID-19 only (314).

Furthermore, several recent case studies have raised concerns regarding the Mtb reactivation in TB-infected subjects following SARS-CoV-2 co-infection. These reports suggest that since the control of both Mtb and SARS-CoV-2 replication depends on cellular immunity, it is possible that the immune dysregulation caused by SARS-CoV-2 or the immunomodulatory therapies used for COVID-19 treatment may increase the risk for TB reactivation (321–326).

Both SARS-CoV-2 and Mtb have immunomodulating potentials to change the outcome of the course of each disease in co-infected patients: SARS-CoV-2 may cause immunosuppression and cytokine storm, which can contribute to the Mtb reactivation (327) and lung tissue damage; Mtb may cause T-cell exhaustion and uncontrolled release of proinflammatory cytokines resulting in lung damage (328, 329), thus potentially contributing to the susceptibility to SARS-CoV-2 infection and to a more severe COVID-19.

In TB-infected individuals, T cells are responsible for Mtb control *via* the granuloma formation. Co-infection with SARS-CoV-2 in these individuals may negatively affect immune regulation in the granuloma leading to Mtb reactivation (322, 330). This alteration of the immune system has been reported using a large-scale meta-analysis of transcriptomic data showing that some immune genes are enriched in COVID-19 and TB diseases (309). The findings from case reports indicate the presence of similarities in the immunopathogenesis of the two diseases, which may exacerbate disease severity during co-infection. Subclinical and clinical TB disease may increase the risk of severe COVID-19 disease and also SARS-CoV-2 co-infection may induce the progression to TB disease (309), as reported above (321–326). In this regard, IFN-I which is strongly induced by viral infection may be detrimental in the context of Mtb by inhibiting B cell responses, inducing the release of immunosuppressive molecules or reducing the macrophagic activation induced by IFN-γ (145), Also, the hyperinflammatory milieu caused by Mtb may raise the risk of severe COVID-19 and *vice versa* (331). Mtb spread or reactivation might be favored by inflammatory molecules released from the SARS-CoV-2-induced necroptosis, whereas the apoptosis might mitigate it (332). Moreover, while COVID-19 therapies targeting pro-inflammatory cytokines may limit the acute immunopathology, they may also repress the responses needed to control Mtb containment (308).

Altogether, these studies suggest that co-infection alters the capacity of the host to respond to and control Mtb and/or SARS-CoV-2, indicating the need for further investigation of the underlying immunological pathways.

## Final remarks

**Figure 3:**
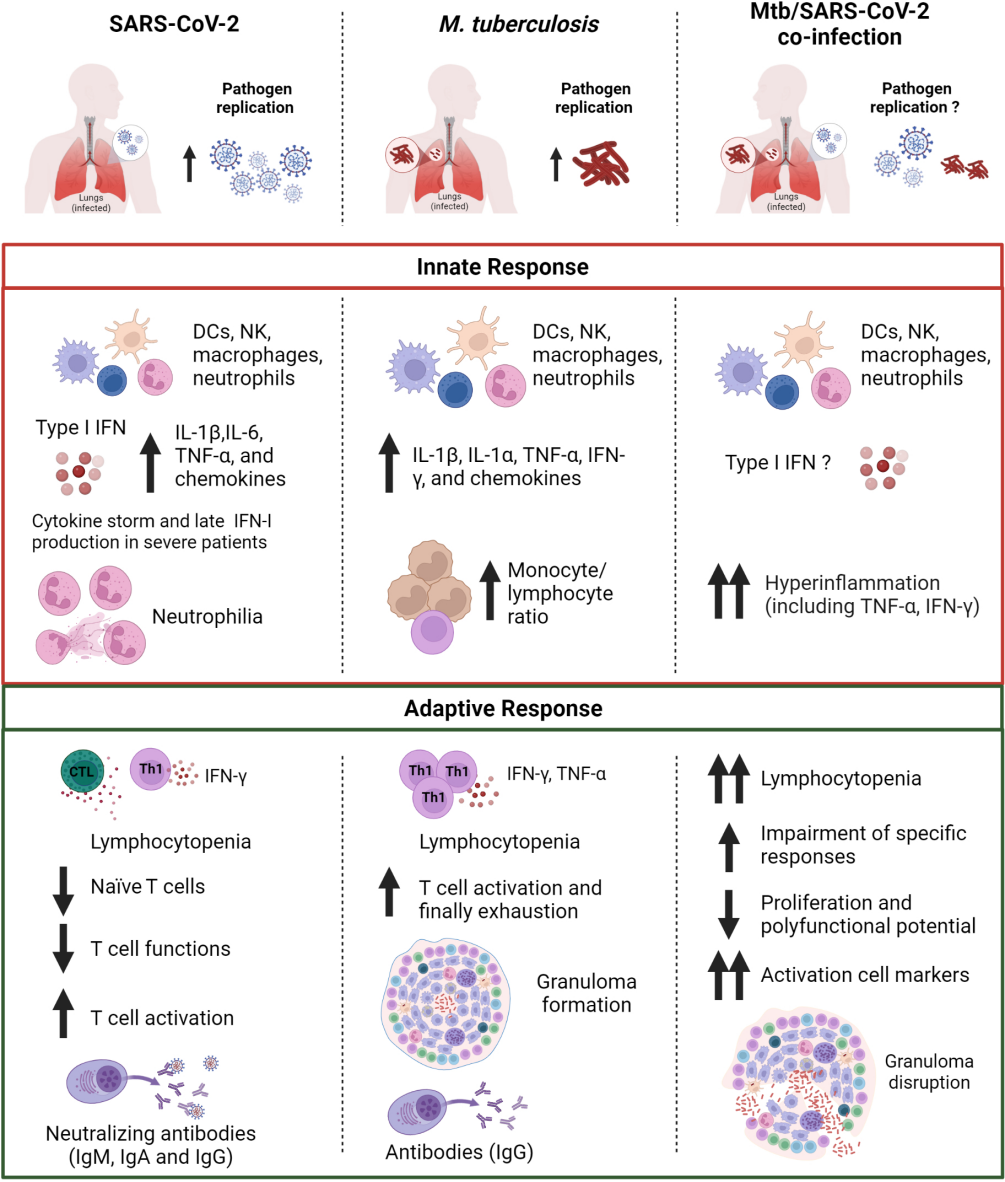
Comparison of the immune response in SARS-CoV-2, Mtb or Mtb/SARS-CoV-2 infection. The innate immune response induced after exposure to SARS-CoV-2 or Mtb is characterized by the production of pro-inflammatory cytokines including IL-1β and TNF-α. In Mtb/SARS-CoV-2 co-infection there is an overproduction of pro-inflammatory cytokines. SARS-CoV-2 infection presents also an early type I IFN production, which is absent or delayed in severe COVID-19 patients. SARS-CoV-2 infection is also characterized by a higher neutrophil count, whereas a higher monocyte/lymphocyte ratio is observed in Mtb-infected patients. Both SARS-CoV-2 and Mtb infected subjects show lymphocytopenia and T cell activation, which are even more prominent in case of co-infection. In co-infected individuals a major impairment of antigen-specific response to Mtb and SARS-CoV-2, and granuloma disruption is present. SARS-CoV-2, severe acute respiratory syndrome coronavirus 2; Mtb, *Mycobacterium tuberculosis*; IFNs, interferons; DCs, dendritic cells; NK, natural killer; Th, T helper; Ig, immunoglobulin. Created with BioRender.com.

SARS-CoV-2 and Mtb are currently the two deadliest infectious diseases in humans. While the route of infection and the target organ are similar, the time to disease manifestation and the pathways driving immunopathology differ significantly (**Figure 3**).

Evidence reported here show that both innate and adaptive immune response are critical components for the protection against SARS-CoV-2 and Mtb. The immune response to both SARS-CoV-2 and Mtb is complex and multifaceted, and there are still many aspects that are not well understood. However, it is known that an appropriate activation of the innate immunity in the early stages of infection followed by adaptive immunity is necessary to curb the pathogen dissemination in the host.

The comparison of these two pathogens highlights how the innate immune response induced after exposure to SARS-CoV-2 or Mtb share the production of some pro-inflammatory cytokines including IL-1β and TNF-α. Similar results were found in Mtb/SARS-CoV-2 co-infection. For SARS-CoV-2 infection, the early and robust IFN-I production as well as neutralizing antibodies have an outmost importance for guarantee an efficient control of viral spread and to determine the clinical outcome of COVID-19. On the other hand, in Mtb infection a central role is played by the alveolar macrophages and the cytokines they release as TNF-α and IL-1β.

Although the infections caused by the individual pathogens have been intensively studied, there are still many unanswered questions about the influence of these pathogens on each other, the immune response, and clinical outcome in the context of co-infection. Recent data has raised concerns regarding the Mtb reactivation following SARS-CoV-2 infection likely due to immune dysregulation caused by SARS-CoV-2 or immunomodulatory COVID-19 therapies. Further clinical and scientific research is needed to better understand the interaction and outcome of the co-infection.

## Author contributions

AA contributed to the writing of introduction, immune response to SARS-CoV-2, final remarks and created the figures. SN-F was responsible for immune response to *M. tuberculosis*, co-infection and tables. DG conceived the review, contributed to the first draft and revised the whole manuscript. All the authors approved the final version of the manuscript.
